# Effects of smoking on delayed neuropsychiatric sequelae in acute carbon monoxide poisoning: A prospective observational study

**DOI:** 10.1097/MD.0000000000026032

**Published:** 2021-05-21

**Authors:** Sangun Nah, Sungwoo Choi, Sun-Uk Lee, Gi Woon Kim, Young Hwan Lee, Sangsoo Han

**Affiliations:** a Department of Emergency Medicine, Soonchunhyang University Bucheon Hospital, Bucheon, Republic of Korea; b Department of Neurology, Korea University Medical Center, Seoul, Republic of Korea.

**Keywords:** carbon monoxide, neuropsychological sequelae, neurotoxicity, poisoning, smoking

## Abstract

Smoking is a well-known risk factor for cardio-cerebrovascular disease. However, several studies have reported the “smoker's paradox” whereby smokers have a better prognosis for cardio-cerebrovascular diseases. Similar to cardio-cerebrovascular diseases, hypoxia is one of the major mechanisms of injury in carbon monoxide (CO) poisoning. This study investigated the association between smoking and delayed neuropsychiatric sequelae (DNS) in acute CO poisoning.

This study involved patients with CO poisoning treated at a university hospital in Bucheon, Korea between September 2017 and March 2020. The exclusion criteria were age <18 years, discharge against medical advice, loss to follow-up, persistent neurological symptoms at discharge, transfer from another hospital 24 hours after exposure, and transfer from another hospital after hyperbaric oxygen therapy. Logistic regression analysis was performed to find factors associated with DNS.

Two hundred sixty three patients visited the hospital due to CO poisoning and of these, 54 were excluded. DNS was evaluated up to 3 months after discharge, and until this time, DNS occurred in 35 (16.8%) patients. And the incidence rate of DNS was lower in smokers than non-smokers (15, 12% vs 20, 23.8%, *P* = .040). Multivariable logistic regression analysis revealed that CO exposure time (odds ratio [OR] 1.003; confidence interval [CI] 1.001–1.005; *P* = .003), the Glasgow coma scale (GCS) (OR 0.862; CI 0.778–0.956; *P* = .005), and pack-years (OR 0.947; CI 0.903–0.993; *P* = .023) were statistically significant for DNS development.

These results indicate that more pack-years smoked were associated with reduced risk of the development of DNS in acute CO poisoning, and that CO exposure time and GCS is a predictive factor for DNS occurrence.

## 1 Introduction

Carbon monoxide (CO) poisoning is one of the leading causes of mortality and morbidity among poisoning accidents, with incidence and mortality rates of 137 and 4.6 per million people worldwide, respectively.^[1,2]^ As carbon monoxide (CO) binds to hemoglobin with affinity about 250 times stronger than that of oxygen, even a small amount of CO poisoning can cause tissue hypoxia by shifting the oxyhemoglobin dissociation curve to the left.^[3]^ Additionally, CO binds to myoglobin and mitochondrial cytochrome oxidase, thus limiting oxygen supply and causing lipid peroxidation.^[4]^ Even a small amount of CO can induce hypoxia and injury to cells, and organs with particularly high metabolic requirements, such as the brain and heart, can be damaged by CO poisoning, resulting in neurological deficits or even death.^[5,6]^

CO poisoning can cause delayed neuropsychiatric sequelae (DNS), defined as brain injury that occurs after a lucid interval of several days to 6 weeks after recovery from hypoxic injury.^[7]^ DNS is characterized by a number of symptoms, such as movement disorder, mood disorder, and/or memory disorder.^[8]^ Previous studies have demonstrated that long duration of CO exposure and intentional suicide attempt affected the development DNS, and have reported poor neurological outcomes.^[9,10]^ However, few studies have focused on the association between smoking and the occurrence of DNS associated with CO poisoning.

According to the World Health Organization, more than 1.1 billion people worldwide currently smoke.^[11]^ Smoking is a risk factor for myocardial and cerebrovascular disease, and is also significantly associated with mortality of coronary artery disease.^[12,13]^ However, several studies have reported the “smoker's paradox” whereby smokers have a better prognosis of cardio-cerebrovascular diseases.^[14–16]^ One reported that the mortality rate in acute myocardial infarct patients who underwent thrombolysis was lower among smokers than among non-smokers.^[17]^ Additionally, smokers had lower mortality rates 30 days and 1 year after ischemic stroke than non-smokers.^[18]^

Similar to cardio-cerebrovascular diseases, hypoxia is one of the major mechanisms of injury in CO poisoning. Therefore, we postulated that smoking may influence the prognosis of CO poisoning patients, and conducted this study to investigate the effects of smoking on DNS in acute CO poisoning patients.

## 2 Materials and methods

### 2.1 Design of study

This investigation was a prospective and observational study using a CO registry with all CO poisoning patients who visited the university hospital emergency department (ED) located in Bucheon, Korea. The study was approved by the institutional review board of Bucheon College of Medicine, Soonchunhyang University (IRB file No. 2020-03-019 from April 23, 2020).

### 2.2 Selection of participants

All patients visiting the ED of our hospital with CO poisoning have been enrolled in the CO registry. Patients who told appropriate histories about CO poisoning or had physical signs such as inhalation injuries or burns, and/or obtained carboxyhemoglobin (COHb) value >5% for non-smokers and >10% for smokers upon the first visit to the ED were regarded as CO poisoning patients. This study was conducted on CO poisoning patients who visited from September 2017 to March 2020. The exclusion criteria were age <18 years, discharge against medical advice, loss to follow-up, persistent neurological symptoms at discharge, transfer from another hospital 24 hours after exposure, and transfer from another hospital after hyperbaric oxygen (HBO) therapy.

### 2.3 Clinical and laboratory assessments

Demographic data, vital signs, medical comorbidities, smoking history, Glasgow coma scale (GCS), CO poisoning intentionality (accidental or intentional), accompanying symptoms, HBO therapy implementation, and laboratory results were collected. Neurological symptoms and signs were also assessed prospectively at discharge. We defined DNS as neurological deficits occurring within 3 months after discharge and consisted of symptoms such as depressed mood, movement disorder, consciousness disturbance, memory disorder, insomnia, headache, dizziness, and Parkinson-like syndrome.^[8,19]^ Patients and family were educated about DNS and discharged with relevant reading materials. One month after discharge, patients visited the ED and emergency medicine specialists evaluated the existence of DNS symptoms. Telephone interviews regarding the development of DNS were also conducted with all patients at 2 weeks, 6 weeks, and 3 months after discharge. When patients were considered to have DNS symptoms, they were re-evaluated to ensure an accurate diagnosis of DNS based on additional examinations, including brain magnetic resonance imaging (MRI) and mini-mental status examination, as well as consultation with neurologists and psychiatrists. After consultation for excluding other causes of neurological deficits and/or psychiatric disorders, DNS was finally diagnosed. The cognitive disorder was defined as a neurologist-administered Mini-mental state examination with a score of <24, and the memory disorder was defined as impaired function of delayed recall of a word list.^[20]^ Parkinsonism was defined as the presence of ≥2 of the symptoms, such as resting tremor, rigidity, bradykinesia, and impairment of postural reflexes. For the evaluation of psychiatric disease, the criteria of the Diagnostic and Statistical Manual of Mental Disorders-5 (DSM-5) was used. Depressed mood was defined as the presence of ≥5 symptoms of the DSM-5.

### 2.4 Management of CO poisoning

All CO poisoning patients received 15 L of oxygen per minute using a non-rebreather mask in the ED before HBO therapy. At our hospital, the indications for HBO therapy in non-pregnant patients include the initial COHb level >25% or the presence of neurological abnormalities regardless of COHb level, and/or there was obvious acute cardiac injury, such as abnormal electrocardiogram or cardiac enzyme level. HBO therapy was applied in a monochamber (IBEX MONO; IBEX Medical Systems Co., Tel Aviv, Israel) and treatment was conducted in 3 sessions at intervals of 6 to 12 hours within 1 day. In the first session, the total duration of HBO therapy was 150 min/session and the target pressure was 3 atmosphere absolute (ATA). Subsequent sessions were for 120 min/session and 2 ATA.^[21]^ According to our management protocol, compression was conducted in the first 30 minutes of each session and decompression was conducted from 30 minutes before the end of each session.

### 2.5 Data analysis

Categorical variables are expressed as absolute numbers or relative frequencies, and continuous variables are reported as the median with interquartile range. Continuous variables with a normal distribution were compared using Student *t* test and those with a non-normal distribution were compared using the Mann–Whitney *U* test. And the normal distribution was examined by a Shapiro-Wilk test. Pearson Chi-squared test and Fisher exact test were used for categorical variables in both non-DNS and DNS (the DNS prevalence was evaluated up to 3 months after discharge), and non-smoker and smoker. Multivariable logistic regression analysis was performed to identify factors related to the occurrence of DNS. Adjusted odds ratios (ORs) and 95% confidence intervals (CIs) were calculated for all statistically significant variables. And 2-tailed *P* < .05 was taken to indicate statistical significance. All statistical analyses were conducted using SPSS for Windows version 26 (IBM, Armonk, NY).

## 3 Results

**Figure 1:**
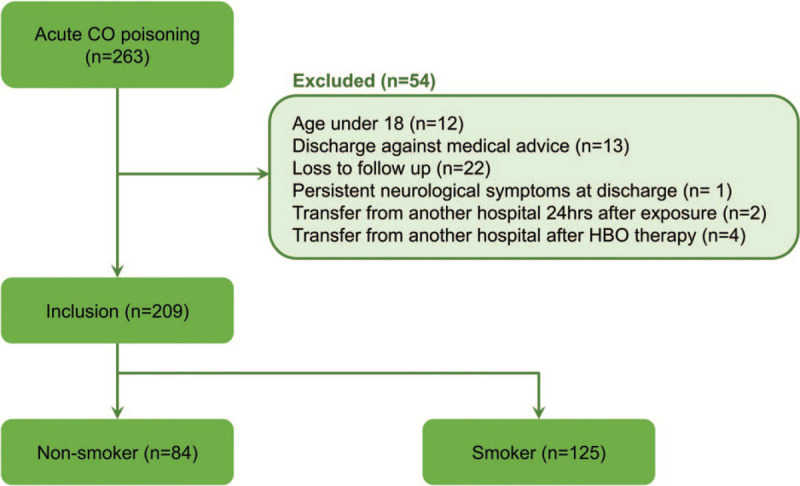
Algorithm for patient selection. CO = carbon monoxide, HBO = hyperbaric oxygen.

In total, 263 patients visited our ED due to acute CO poisoning. Of these, 54 were excluded for the following reasons: 12 were <18 years old, 13 were discharged against medical advice, 22 were lost to follow-up, 1 had persistent neurological symptoms at discharge, 2 were transferred from another hospital 24 hours after exposure, and 4 were transferred from another hospital after HBO therapy (Fig. 1). Therefore, 209 patients were finally included in the study.

The baseline characteristics of the patients are listed in Table 1. The median age was 43, and 142 (67.9%) were men. The study population included 125 (59.8%) current smokers and 150 (71.8%) had intentional exposure to CO. The median number of pack-years was 10, initial GCS score was 15, and DNS occurred in 35 (16.8%) patients.

**Table 1:** Baseline characteristics of the study population.

	Total (n = 209)
Age, y	43 [33–54]
Male (%)	142 (67.9)
BMI, kg/m^2^	23.2 [21.0–25.6]
Underlying disease (%)	
Hypertension	19 (9.1)
Diabetes	10 (4.8)
Current smoker (%)	125 (59.8)
Pack-years^∗^	10 [7.5–20]
Intentional exposure (%)	150 (71.8)
Initial GCS	15 [11–15]
CO exposure time, min	187 [120–240]
Performed HBOT (%)	185 (88.5)
Occurrence of DNS (%)	35 (16.8)

Table 2 lists the differences in characteristics between non-smokers and smokers. The proportions of men differed significantly between groups: 101 (80.8%) in the smoking group and 41 (48.8%) in the non-smoking group. Initial GCS scores and CO exposure times did not differ between the 2 groups. The rate of intentional exposure to CO was significantly higher for smokers than non-smokers (97, 77.6% vs 53, 63.1%, respectively). With regard to laboratory findings, the rates of COHb were 11.3% in smokers and 9.4% in non-smokers, and lactate concentrations were 2.6 mmol/L in smokers and 1.7 mmol/L in non-smokers, and both of these values were significantly higher in smokers. The rate of DNS was lower in smokers than non-smokers (15, 12% vs 20, 23.8%). Contrary to Table 2, initial GCS scores and CO exposure times were statistically different and the non-DNS group's number of pack-years were more than DNS group (Table S1, Supplemental Digital Content).

**Table 2:** Comparison of baseline characteristics between smokers and non-smokers.

	Non-smoker	Smoker	*P*-value
	(N = 84)	(N = 125)	
Age, y	46 [35–58]	42 [31–50]	.098
Male, n (%)	41 (48.8)	101 (80.8)	<.001^∗^
BMI	22.8 [20.7–25.4]	23.4 [21.2–25.7]	.337
Underlying disease (%)			
Hypertension	7 (8.3)	12 (9.6)	.947^∗^
Diabetes	4 (4.8)	6 (4.8)	>.99^∗∗^
Vital signs			
Systolic BP, mm Hg	130 [120–140]	130 [115–140]	.966
Diastolic BP, mm Hg	80 [70–90]	80 [80–90]	.220
Heart rate, /min	90 [78–100]	95 [85–105]	.018
Respiratory rate, /min	20 [19–20]	20 [18–20]	.855
Oxygen saturation, %	98 [97–98]	98 [95–98]	.320
Initial GCS	15 [12.8–15]	15 [10–15]	.846
CO exposure time, min	187 [120–255]	187 [90–240]	.262
Performed HBOT	80 (95.2)	105 (84.0)	.023^∗^
Intentional exposure (%)	53 (63.1)	97 (77.6)	.033^∗^
Symptoms (%)			
Headache	10 (11.9)	12 (9.6)	.762^∗^
LOC	28 (33.3)	33 (26.4)	.354^∗^
Dizziness	11 (13.1)	17 (13.6)	>.99^∗^
Dyspnea	4 (4.8)	5 (4.0)	>.99^∗∗^
Chest pain	3 (3.6)	4 (3.2)	>.99^∗∗^
Laboratory findings			
COHb, %	9.4 [3.4–14.3]	11.3 [5.4–19.7]	.069
WBC, ×10^3^/mm^3^	11.7 [8.6–15.2]	12.1 [8.2–15.6]	.900
BUN, mg/dL	14.8 [11.2–19.4]	12.9 [10.8–17.7]	.111
Creatinine, mg/dL	1 [0.9–1.2]	1 [0.9–1.2]	.077
Creatine kinase, U/L	143 [83–520]	121 [86.5–266.5]	.323
Arterial pH	7.4 [7.4–7.5]	7.4 [7.4–7.4]	.139
CRP, mg/dL	0.1 [0.1–0.4]	0.1 [0.1–0.4]	.286
Lactate, mmol/L	1.7 [1.2–3.3]	2.6 [1.7–4.6]	.044
Myoglobin, ng/mL	56 [26.5–385.0]	39.4 [25–244]	.319
Troponin I, ng/mL	0.1 [0.1–0.3]	0.1 [0.1–0.1]	.755
CK-MB, ng/mL	3.3 [1.6–16.1]	1.95 [1.2–4.6]	.007
Occurrence of DNS	20 (23.8)	15 (12.0)	.040^∗^

**Figure 2:**
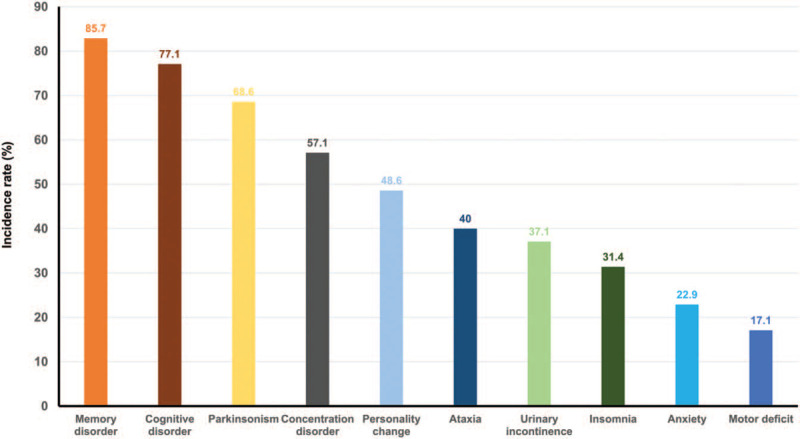
Distribution of neurological abnormalities of DNS in CO poisoning patients. The percentages are relative to the cohort of those with 35 patients who have DNS. CO = carbon monoxide, DNS = delayed neuropsychiatric sequelae.

Neurological abnormalities in patients who developed DNS were as follows: memory disorder (29 patients), cognitive disorder (27 patients), Parkinsonism (24 patients), concentration disorder (20 patients), personality change (17 patients), ataxia (14 patients), urinary incontinence (13 patients), insomnia (11 patients), anxiety (8 patients), and movement disorder (6 patients) (Fig. 2, Table 3). Also, comparison of neurological symptoms in patients who developed DNS between the smokers and non-smokers can be identified in Table 3.

**Table 3:** Comparison of neurological symptoms and signs in patients who developed delayed neuropsychiatric sequelae between the smokers and non-smokers.

	Total	Non-smoker	Smoker	
	(N = 35)	(N = 20)	(N = 15)	*P*-value
Memory disorder (%)	29 (82.9)	17 (85.0)	12 (80.0)	>.999^∗∗^
Cognitive disorder (%)	27 (77.1)	14 (70.0)	13 (86.7)	.419^∗∗^
Parkinsonism (%)	24 (68.6)	14 (70.0)	10 (66.7)	>.999^∗∗^
Concentration disorder (%)	20 (57.1)	13 (65.0)	7 (46.7)	.460^∗^
Personality change (%)	17 (48.6)	10 (50.0)	7 (46.7)	>.999^∗^
Ataxia (%)	14 (40.0)	8 (40.0)	6 (40.0)	>.999^∗^
Urinary incontinence (%)	13 (37.1)	8 (40.0)	5 (33.3)	.960^∗^
Insomnia (%)	11 (31.4)	6 (30.0)	5 (33.3)	>.999^∗∗^
Anxiety (%)	8 (22.9)	6 (30.0)	2 (13.3)	.419^∗∗^
Motor deficit (%)	6 (17.1)	4 (20.0)	2 (13.3)	.680^∗∗^

Univariable logistic regression analysis of DNS development revealed significant differences in GCS scores and pack-years between the 2 groups (*P* < .05): CO exposure time (OR 1.003; CI 1.001–1.005; *P* = .001), GCS (OR 0.878; CI 0.795–0.969; *P* = .010), and pack-years (OR 0.953; CI 0.910–0.998; *P* = .040). Multivariable logistic regression analysis also revealed that CO exposure time (OR 1.003; CI 1.001–1.005; *P* = .003), GCS (OR 0.873; CI 0.786–0.971; *P* = .012), and pack-years (OR 0.954; CI 0.910–1.000; *P* = .049) were statistically significant (Table 4).

**Table 4:** Univariable and multivariable logistic regression analyses of risk factors for DNS associated with CO poisoning.

	Univariable		Multivariable	
	Odds ratio	*P*-value	Odds ratio	*P*-value
Age, y	1.014 (0.990–1.039)	.264		
Male, n (%)	0.492 (0.234–1.033)	.061		
BMI	0.982 (0.887–1.086)			
Comorbidities				
Hypertension	1.368 (0.425–4.399)	.599		
Diabetes	0.536 (0.066–4.370)	.560		
Vital signs				
Systolic BP, mmHg	0.999 (0.980–1.018)	.911		
Diastolic BP, mmHg	1.007 (0.981–1.033)	.620		
Heart rate, /min	0.995 (0.974–1.016)	.622		
Respiratory rate, /min	0.921 (0.759–1.118)	.405		
Oxygen saturation, %	1.019 (0.941–1.103)	.648		
Performed HBOT	1.464 (0.412–5.204)	.556		
Symptoms				
Headache	0.467 (0.104–2.094)	.320		
LOC	1.33 (0.615–2.881)	.468		
Dizziness	0.559 (0.159–1.964)	.364		
Dyspnea	0.610 (0.074–5.040)	.647		
Chest pain	0.824 (0.096–7.056)	.859		
Laboratory findings				
COHb, %	0.995 (0.964–1.027)	.745		
WBC, ×10^3^/mm^3^	0.998 (0.93–1.072)	.966		
BUN, mg/dL	1.016 (0.970–1.064)	.496		
Creatinine, mg/dL	1.17 (0.910–1.505)	.221		
Creatine kinase, U/L	1.000 (1.000–1.000)	.222		
Arterial pH	0.613 (0.005–78.331)	.843		
Myoglobin, ng/mL	1 (1.000–1.000)	.670		
Troponin I, ng/mL	1.389 (0.873–2.209)	.166		
CK-MB, ng/mL	1.006 (0.999–1.013)	.079		
CRP, mg/dL	1.076 (0.975–1.189)	.146		
Lactate, mmol/L	0.476 (0.187–1.211)	.119		
CO exposure time, min	1.003 (1.001–1.005)	.001	1.003 (1.001–1.005)	.003
GCS	0.878 (0.795–0.969)	.010	0.873 (0.786–0.971)	.012
Pack-years^∗^	0.953 (0.910–0.998)	.040	0.954 (0.910–1.000)	.049

## 4 Discussion

This study investigated DNS in current smokers exposed to CO. DNS occurred in 35 (16.7%) of 209 patients who visited the hospital due to CO poisoning, and our results indicated that greater number of pack-years was related to reduced risk of the development of DNS in acute CO poisoning. To our knowledge, this is the first report of an association between number of pack-years of smoking and incidence of DNS in acute CO poisoning patients.

The mechanism of DNS has not been clearly elucidated. In previous studies, DNS was reported to occur through pathophysiological mechanisms such as brain tissue hypoxia, increase in reactive oxygen species (ROS) due to ischemia–reperfusion injury, and immunological reactions, and its incidence is reported to range from about 3% to 40%.^[6,22,23]^ In the present study, the incidence of DNS was 16.7%, which was consistent with previous reports. Jeon et al^[24]^ and Pepe et al^[23]^ reported that memory disorder was the most common DNS symptom. In our study also, memory disorder was reported the most common. There were no significant difference in DNS symptoms between the 2 groups of smoking and non-smoking.

There have been conflicting reports regarding the correlation between GCS and DNS. Pepe et al^[23]^ studied the predictive value of GCS for DNS in 347 CO poisoning patients (including voluntary and accidental exposure), and reported a significant correlation between low GCS score and DNS. Kitamoto et al^[9]^ conducted a similar study in 88 CO poisoning patients and obtained the similar results. However, in a study of 138 CO poisoning patients, Lee et al^[25]^ reported that GCS was not associated with the occurrence of DNS. We found a correlation between DNS and low GCS score, which is consistent with the former 2 reports.

**Figure 3:**
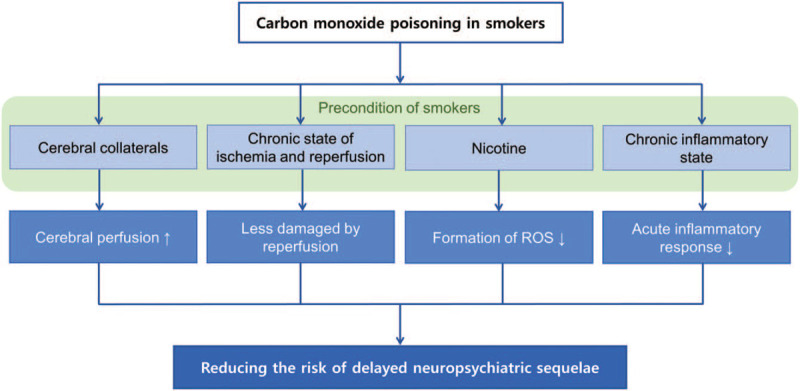
Theory on the pathophysiology of smoking's neuroprotective effect in CO poisoning. CO = carbon monoxide, ROS = reactive oxygen species.

Smoking causes an increase in hematocrit, platelet activation and aggregation, vasoconstriction, increase in circulating fibrinogen, thrombin production, and endogenous fibrinolytic damage.^[18]^ Therefore, the risks of cardio-cerebrovascular diseases are increased in smokers.^[14,15]^ However, smokers also have a better prognosis in cases of cardio-cerebrovascular diseases, which is referred to as the “smoker's paradox.”^[16]^ In the present study, smokers had a better neurological prognosis than non-smokers; we also found that smokers with many pack-years of smoking are at lower risk of developing DNS. The pathophysiology of DNS is known to involve hypoxia-induced injury and ischemia–reperfusion injury as described above.^[6]^ Therefore, smoking may have a neuroprotective effect through the following mechanisms (Fig. 3). First, smoking induces ischemic preconditioning by causing patients to undergo chronic changes in vasomotor tone and episodic hypoxia, and can improve cerebral perfusion by activating the cerebral collaterals.^[18]^ Second, current smokers continuously suffer ischemia and reperfusion, so in the event of ischemic diseases, such as myocardial infarction (MI), the cells are paradoxically less damaged by reperfusion during recovery.^[26]^ Third, cigarettes contain nicotine, which has neuroprotective effects.^[28]^ Ischemia–reperfusion injury involves the overproduction of ROS by mitochondria, resulting in cell injury.^[27]^ Nicotine limits the formation of ROS by inhibiting the binding of nicotinamide adenine dinucleotide to complex I.^[28]^ Fourth, smokers are in a chronic inflammatory state known as inflammatory preconditioning, which reduces platelet activity and chemotaxis of inflammatory cells and induces microvascular dysfunction.^[29]^ Due to this mechanism, the acute inflammatory response is reduced and the injury to the cells is decreased. Therefore, there may be differences in the incidence of DNS in smokers, because DNS is also an inflammatory reaction caused by acute CO poisoning.^[30]^

Despite these remarkable findings, this study had several limitations. First, it was a single-center study, so further larger multicenter prospective studies are needed to obtain more reliable results. Second, we investigated the association between smoking and DNS; more research is needed to investigate this association in populations of CO poisoning patients who are ex-smokers. Third, the use of electronic cigarettes has become common, and body COHb is increased to a lesser extent with electronic cigarettes compared with conventional cigarettes.^[31]^ We did not differentiate between electronic and conventional cigarettes, so it was not possible to know how this classification affected DNS. Fourth, the average half-life of nicotine is about 2 hours in the human body, and the terminal half-life has been reported to be up to 17 hours.^[32]^ However, nicotine concentration was not measured at the time of CO exposure, so we could not determine the differences in DNS occurrence according to nicotine concentration. Fifth, because 20% of patients were excluded from analysis, the relationship between DNS and risk factor may be affected. Sixth, caution is needed to generalize our results. According to a recent study, smokers with MI who underwent primary percutaneous coronary intervention were associated with worse prognosis in long term.^[33]^ Therefore, further research is also needed on the “smoker's paradox” in CO poisoning.

## 5 Conclusion

The results of this study revealed that the number of pack-years of smoking is a predictor of the occurrence of DNS in acute CO poisoning. We also found that CO exposure time and GCS can predict the occurrence of DNS. Smoking is a factor that can be easily identified by history taking, so these findings will be useful to clinicians treating acute CO poisoning patients.

## Author contributions

**Conceptualization:** Gi Woon Kim, Sangsoo Han.

**Formal analysis:** Sangun Nah, Sungwoo Choi.

**Investigation:** Sangun Nah, Sungwoo Choi.

**Methodology:** Sun-uk Lee, Young Hwan Lee, Sangsoo Han.

**Supervision:** Sangsoo Han.

**Writing – original draft:** Sangun Nah.

**Writing – review & editing:** Sangsoo Han.

## Supplementary Material

Supplemental Digital Content
