# Supplementary material for: Effects of smoking on delayed neuropsychiatric sequelae in acute carbon monoxide poisoning: A prospective observational study
Source: Medicine (Baltimore). 2021 May 21;100(20):e26032. doi: 10.1097/MD.0000000000026032 (PMC8137110; doi:10.1097/MD.0000000000026032)
Supplement: Supplemental Digital Content [file medi-100-e26032-s001.docx]

Table S1. Comparison of baseline characteristics between the non-DNS and DNS groups

|  | **Non-DNS** | **DNS** | **p-value** |
| --- | --- | --- | --- |
|  | **(N=174)** | **(N=35)** |  |
| Age, years | 42.5 [32-53.8] | 44 [35-57] | .244 |
| Male, n (%) | 123 (70.7) | 19 (54.3) | .089* |
| BMI | 23.2 [21.1-25.7] | 23.1 [19.9-25.4] | .486 |
| Underlying disease (%) |  |  |  |
| Hypertension | 15 (8.6) | 4 (11.43) | .532** |
| Diabetes | 9 (5.2) | 1 (2.9) | >.99** |
| Current smoker (%) | 110 (63.2) | 15 (42.9) | .040* |
| Pack-years | 6 [0-12.88] | 0 [0-8.75] | .022 |
| Vital signs |  |  |  |
| Systolic BP, mmHg | 130 [117.8-140] | 130 [117.5-140] | .962 |
| Diastolic BP, mmHg | 80 [76-90] | 80 [70-90] | .913 |
| Heart rate, /min | 92 [80-102.8] | 90 [77.5-103] | .502 |
| Respiratory rate, /min | 20 [18.3-20] | 20 [18.5-20] | .541 |
| Oxygen saturation, % | 98 [96-98] | 98 [95.5-98.5] | >.99 |
| Initial GCS | 15 [12-15] | 13 [8-15] | .011 |
| CO exposure time, min | 187 [97.5-187] | 360 [187.5-394] | <.001 |
| Performed HBOT | 153 (87.9) | 32 (91.4) | .773** |
| Intentional exposure (%) | 123 (70.7) | 27 (77.1) | .570* |
| Symptoms (%) |  |  |  |
| Headache | 20 (11.5) | 2 (5.7) | .544** |
| LOC | 49 (28.2) | 12 (34.3) | .601* |
| Dizziness | 25 (14.4) | 3 (8.6) | .585** |
| Dyspnea | 8 (4.6) | 1 (2.9) | >.99** |
| Chest pain | 6 (3.4) | 1 (2.9) | >.99** |
| Laboratory findings |  |  |  |
| COHb, % | 10.5 [4.3-17.7] | 9.4 [2.5-18.4] | .417 |
| WBC, ×10^3^/mm^3^ | 12.0 [8.2-15.5] | 10.9 [8.6-15.8] | .842 |
| BUN, mg/dL | 13.5 [11.1-17.9] | 15.1 [11.1-18.5] | .330 |
| Creatinine, mg/dL | 1 [0.9-1.2] | 1 [0.9-1.1] | .578 |
| Creatine kinase, U/L | 124 [85-281] | 181 [85.5-737.3] | .205 |
| Arterial pH | 7.4 [7.4-7.4] | 7.4 [7.4-7.4] | .720 |
| CRP, mg/dL | 0.1 [0.04-0.3] | 0.4 [0.1-0.8] | .007 |
| Lactate, mmol/L | 2.4 [1.6-4.3] | 1 [0.9-1.9] | .019 |
| Myoglobin, ng/mL | 45 [25.3-230.5] | 59 [27-364] | .533 |
| Troponin I, ng/mL | 0.1 [0.1-0.2] | 0.1 [0.1-0.4] | .469 |
| CK-MB, ng/mL | 2.2 [1.4-5.8] | 3.5 [1.6-32.5] | .079 |

Notes: Values are expressed as the median [interquartile range], or number (proportion). *Pearson’s χ^2^ test, ** Fisher’s exact test.

Abbreviations: DNS, delayed neuropsychiatric sequelae; BMI, body mass index; BP, blood pressure; GCS, Glasgow coma scale; CO, carbon monoxide; HBOT, hyperbaric oxygen therapy; LOC, loss of consciousness; COHb, carboxyhemoglobin; WBC, white blood cells; BUN, blood urea nitrogen; CRP, C-reactive protein; CK-MB, creatine kinase-myocardial band.
